# Developing a Framework to Support the Delivery of Effective Pain Management for Children: An Exploratory Qualitative Study

**DOI:** 10.1155/2020/5476425

**Published:** 2020-10-28

**Authors:** Joan Simons, Bernie Carter, Jennie Craske

**Affiliations:** ^1^The Open University, Milton Keynes, UK; ^2^Edge Hill University, Ormskirk, UK; ^3^Alder Hey Children's NHS Foundation Trust, Liverpool, UK

## Abstract

Two million children are admitted to hospital every year in the UK and between 59% and 94% will experience pain, with 27–40% of them experiencing moderate to severe pain. Currently, there are a number of well-researched guidelines on children's pain available, yet pain prevalence is high. Despite the guidelines, there is a lack of an overall framework that includes the necessary components to deliver effective pain management. This study was built on previous work about key elements that support children's pain management, by exploring their relevance and practical application with 43 healthcare practitioners. We carried out focus groups with band 5 nurses (*n* = 6) and advanced nurse practitioners (*n* = 11) and semistructured interviews with pain nurses (*n* = 16) and consultants (*n* = 10). We also presented and discussed our findings with an advisory group. Findings demonstrated that the following elements were considered to be important: delivering pain management with confidence, supporting colleagues with protocols and guidance, empowering parents to be involved in pain management, and adopting an individual approach to a child and family. These elements formed the basis of a framework for children's pain management. Some practitioners indicated that pain management required education and more resources, and that the culture of an area could influence pain management practice. The framework brings together elements that have the potential to improve the management of children's pain through its use as an education tool. Each interrelated element of the framework plays an important part in the overall management of children's pain. The need now is to make the dissemination of the findings accessible to health care practitioners, parents, and educators. Next steps include the development of infographic posters, an animation, and a free online course, which will incorporate the use of Bloom's taxonomy.

## 1. Background and Literature Review

There are two million hospital admissions of children in England annually [1]. Studies report a pain prevalence of 59%–94% among hospitalised children [1–3] with 27%–40% of them experiencing moderate to severe pain [3, 4]. Two well-evidenced guidelines developed by the Royal College of Nurses [5] and the Association of Paediatric Anaesthetists [6] are available to guide how pain management should be delivered to children in the UK. The approach of these guidelines is operational in nature, instructing individual practitioners how to assess pain and deliver effective interventions to relieve pain. Despite guidance on how to effectively manage children's pain [5–7], these guidelines and directives do not effectively or consistently change practice [8]. Children's pain management can be opportunistic, simplistic, and frequently interrupted, and children in hospital still experience unnecessary pain [9]; this can have long lasting negative consequences.

Good communication, effective information sharing, and clarity about roles can all positively influence the effectiveness of pain management. However, nurses may not take as active a role as they could do in managing children's pain, seeing it as the parents' and child's responsibility to inform them when they are experiencing pain [10]. Sharing information with parents and valuing their knowledge of their child and communicating in an equal partnership can establish parents' expectations of involvement in the management of their child's pain [11–13]. However, a lack of communication and information provision and poor negotiation of roles can result in missed opportunities for nurses to work in partnership with parents even when parents attempt to be involved and advocate for their child's pain care [11–13].

A study by Simons [14] of 28 practitioners in the UK, Sweden, and Australia explored innovations in pain management practice. The findings identified five key elements that contributed to the delivery of effective pain management to children in hospital; these being

- Distributed pain management with vision
- Effective pain management with less stress
- Delivered with confidence
- Individual approach to child and parent
- Raising parents' expectations of effective pain management

These elements identify the areas that need to be considered by all stakeholders (nurses, other health practitioners, and parents) in the absence of a framework or model of pain management.

## 2. Methods

The study aimed to critically explore practitioners' views of five key elements of pain management and their relevance as a framework for children's pain management and potential for implementation into practice.

The study used an interpretive, exploratory qualitative two-phase design using focus groups (Phase 1) and interviews (Phase 2), which were carried out either face-to-face or remotely. This design was appropriate as it afforded the nurse researchers the opportunity to focus on gaining a deeper understanding [15] of practitioners' views and to gain insight into what benefits an overarching framework for pain management could offer and its implications for the management of children's pain.

### 2.1. Sampling and Recruitment

A mixture of convenience (Phase 1) and purposive sampling (Phase 2) was used. In Phase 1, two groups of nurses working at one tertiary children's hospital and attending specific in-house pain education days were targeted. This included band 5 nurses with at least one year's experience of working within a children's ward and who manage children's pain on a day-to-day basis and advanced nurse practitioners (ANPs) who are nurse prescribers and who have worked as an ANP for more than six months. Phase 2 aimed to recruit experts in pain management (Pain Consultants and Clinical Nurse Specialists) via email invitations to the leading consultant and nurse from each of 17 specialist pain teams in the UK and Ireland via a specialist network (Paediatric Pain Travelling Club), which represents pain teams across the UK and Ireland.

### 2.2. Data Collection

Data were collected in two phases. In both phases, the relevance, implications for practice, and responses to each of the five elements of the five key elements of pain management were explored.

Phase 1 involved focus groups, which were chosen as they allowed us to be time efficient and to gain a wide range of perspectives from nurses in a setting where their ideas could be shared and perspectives bounced off each other. Two focus groups were run at a tertiary children's hospital in the UK.

Phase 2 involved individual face-to-face or remote interviews with pain specialists as this created the opportunity for in-depth discussion that built on the analysis of findings from Phase 1.

Both the focus groups and the interviews were guided by semistructured schedules of questions and prompts that explored issues relating to the five key elements (supplementary files 1 and 2).

An advisory group of six parents and educators supported the study, and of these, one parent and one educator were able to attend a meeting to discuss the findings.

The focus groups and interviews were audio-recorded with permission and transcribed. Notes were taken at the advisory group meeting.

### 2.3. Ethical Considerations

The study gained ethics approval via the NHS Research Ethics Service (IRAS 249555) and from the Open University (HREC/2904/SIMONS).

All participants were provided with a participant information sheet prior to being invited to participate in the study and gave informed written consent. Care was also taken in relation to governance issues (e.g., anonymisation and data protection).

### 2.4. Data Analysis

All three researchers were involved throughout the thematic analysis of focus groups and interviews. Our thematic analysis was undertaken in line with the approach advocated by Braun and Clarke [16, 17]. The analysis of focus groups was used to inform the schedule for Phase 2 interviews. The same process of analysis was used for focus groups and interviews. Each participant's data were analysed as an individual dataset before considering all transcripts as a complete dataset. Initially, each researcher worked independently with the data before working together. We read and reread the transcripts, and initial codes were applied and iteratively considered. Although we were alert for new themes, we were also guided by our a priori themes, which focused on the five key elements of interest. Once each researcher had completed their initial analysis, detailed discussion then took place within the team, and further analysis took place until a broad understanding and consensus was achieved, and the final codes and themes were agreed. The final stage of analysis involved synthesising the findings from focus groups and interviews. No findings were identified, which did not fit within the existing five elements.

## 3. Findings

In total, 43 practitioners were recruited to the study. In Phase 1, 17 nurses (of whom 6 were band 5 nurses and 11 were ANPs) participated in two focus groups. In Phase 2, 26 pain specialists (of whom 10 were consultants and 16 were clinical nurse specialists) participated in face-to-face (*n* = 13) and remote (*n* = 13) interviews across 17 pain teams in the UK and Ireland.

Five themes are presented that represent the findings from both focus groups and interviews; education was a thread that ran across the elements.

In order to protect the identity of participants, anonymised quotations reported in the paper are identified only as being band 5 nurse (N), advanced nurse practitioner (ANP), consultant doctor or anaesthetist (C), and pain nurse (PN).

### 3.1. Distributed Pain Management Leadership with Vision

Some participants were unsure what was meant by “*distributed leadership*,” responding by saying that *“it's not something I am familiar with” (PN)* or it was *“difficult to know what it means”* (C) and needed the concept to be explained. However, once clear about the concept, most participants agreed that it was a good idea with some suggesting pain is “*everyone's business”* (PN) and distributed leadership would *“certainly be the ideal”* (C) as it would generate a stronger feeling of *“buy-in … giving people a voice and letting them come back to you….”* (PN) and ownership of creating change:

*“I think if you have ownership you feel more empowered and then the patients will get better care”* (PN).

It was noted that ANPs felt they were part of *“distributed leadership of effective pain management”* (ANP). A number of practitioners considered education is *“quite key”* and that distributed leadership is *“what we try to achieve with lots of education”* (C) acknowledging the importance of the “*ongoing ability for people to update*” (C) in order to play their role as leaders.

### 3.2. Effective Pain Management with Less Stress

Participants agreed that managing children's pain could be stressful for the children, their parents, and practitioners. Some participants reported stress related to limited resources such as *“No time to spend time with the child and families. No time to listen”* (PN), although some participants had time enough to spend with patients. However, stress could be reduced *“by being there, being approachable”* (PN) and improving access to protocols, dosing guidance, and training at induction. Some participants talked of the importance of *“having good plans in place, so that people feel empowered”* (C) and that the use of *“guidelines and things like that can help reduce stress”* (PN).

Appearing or feeling ineffective could be stressful for staff:

*“Staff get stressed when they look powerless in front of patients and relatives”* (C).

The ANPs noted that support was available to help with the stress of pain management:

*“support of the pain team, alongside protocols and procedures result in less stress and an increase in confidence”* (ANP).

Stress could also be reduced if good interventions were available for every individual child and being able to:

*“provide the right answers or the best answers that you can, or the best care and practice and alter things to reduce that stress”* (PN).

Most participants talked of the importance of keeping children and their families informed as this could help mitigate stress related to pain management:

*“discussing with the families ahead of a painful episode and letting them know what to expect… people available… people assessing the pain management, and ….make adjustments if required”* (C).

Avoiding delays and *“making sure that things are available in a timely manner”* (PN) were identified by participants as being important in both preventing and reducing stress as would using *“the easiest route for administration of medication”* (PN).

A final perspective addressed the value of *“non-pharmacological techniques and psychological therapies” (PN)* and *“trying to encourage them to think of other things”* (PN) as potentially empowering ways of children and parents self-managing stress.

### 3.3. Pain Management Delivered with Confidence

Participants talked of the factors that can influence practitioner confidence, such as experience as *“the more you do it the more confident you get”* (N). Key factors included “*the experience, the education, device training as well”* (C) noting that

*“You can't be confident with your pain delivery unless you've had the training to know that what you're doing is right”* (PN).

Suboptimal prescription and dosage was linked to a lack of confidence with participants noting that *“there's a tendency to under-dose, and as I say it is a confidence issue”* (C), *“giving less paracetamol”* (C) and *“in terms of morphine, people are still very, can be very cautious with it”* (C). Knowing that your actions were *“right”* was fundamental, and confidence came with experience and *“knowing the norm” (PN)*. Knowing *“there's a plan”* (C) supported confident working practices and was *“reassuring”* especially when plans were underpinned by *“readily, easily accessible”* (C) and *“clear easy to understand guidelines and protocols”* (C). Participants talked of the need for confident delivery to be underpinned by good communication and for people to understand the impact of their “*use of language and their demeanour… on patients”* (C). Families *“need to have confidence in the nurse”* (PN), and this confidence could also be facilitated by working with the support of the pain team's expertise when *“the basic package...doesn't fit”* (C).

### 3.4. Individual Approach to Child and Parent

It was noted that *“parents lose confidence if their child is left in pain”* (PN), and there was a universal sense that *“everyone's different, every family is different and every social circumstance is different”* (C). Most practitioners agreed that an individual approach was an important aspect of managing children's pain, acknowledging that *“it should be paramount in all aspects of care, but certainly within pain management”* (PN). This meant that even when working with a protocol, *“tailoring it to that child or family”* (PN) was important, meaning that flexibility was important:

“We won't rigidly stick with a regime just because that's the regime that we do for X. So, I think we're quite flexible” (C).

Noting that *“we have a plan there. But within the plan there could be plan A, B and C depending on the individual child”* (PN). Participants talked of the importance of both *“involving them (child and family) in the conversations at the bedside”* (PN) and treating the *“family as a whole, making sure that they have plenty of information so that they know what to expect”* (C).

One consultant used the expression *“sent to try you”* about the frustration of trying to individualise care in difficult cases but being constrained by hospital systems to complying with the guidelines (that they wrote) aimed at supporting nonexpert practice.

### 3.5. Raising Parents' Expectations of Effective Pain Management

Parents' expectations “*may not be voiced, may not be asked*” (C), suggesting that there is first a need to “*understand”* parents' expectations. Some participants expressed reservations about the possibility of setting unrealistic parental expectations, such as reducing pain to zero, that would then be difficult to meet. However, most thought that sharing realistic expectations was helpful, explaining that *“use of pain pathway helps with parents' expectations of how their child's pain will be managed”* (N). Participants felt that parents' expectations varied between *“think their child will be in pain and they think it's normal”* (C) and *“expect their child won't have any pain or shouldn't have any pain”* (C). So, although *“we can't get rid of all pain at all times”* (PN), there should be a commitment to *“try and sort it out”* (ANP).

Participants reflected that *“parents' understanding (of pain) should be checked, not assumed”* (N) and noted that a *“lack of assertiveness on the part of parents leads to frustration and perhaps avoidable poor pain management”* (N), although parents should not be blamed for this. Parents' expectations could be raised and managed through pain plans and outlining *“how pain will be dealt with [and] be realistic and honest with parents” (PN)* and *“educating them that pain management is not just about the drugs”* (C). Ways of raising expectations included education and communication giving parents “permission” to ask about pain and stressing the need for nurses to talk to parents, for example:

“Pre-operative information for parents is seen as important to start the cycle of effective pain management. This can reduce the potential for stress and clarify expectations—which would lead to a good start to the admission” (N).

Other ways of raising expectations included signs or *“posters or something in the parents' room”* (PN) to prompt parents to *“contact the pain team if you feel your child's pain isn't as well controlled”* (PN).

A consistent approach was deemed important in meeting or raising expectations although it was agreed that this was often dependent on the *“individual nurses on the floor on that particular day unfortunately”* (PN). A consultant reported that *“We can never over-deliver on pain management”* (C)—suggesting that the onus of responsiblity is on the clinicians, rather than the parents with regards to setting the standard of care.

### 3.6. Perspectives on the Elements and Their Potential Implementation via a Framework

Overall, the participants responded positively to the five elements. The following components were ones they thought were important: empowering parents, adopting an individual approach, delivering with confidence, and supporting staff with guidance and protocols. Although some elements were critiqued, overall, it offered something concrete, a *“framework”* (C), something that:

“you can take it to senior management and say “right OK I want to really work on our current pain management and the model of care that we do, and this is what I want to follow” ….. I think that's hugely helpful” (PN).

To be successful, each of the interrelated elements needs to be clearly understood, and all need to work together as a whole. Suggestions for improvement were presented including making it “*more interpretable for band 5 nurses*” (ANP) and including “*more information and examples from practice to explain how each stage could be promoted*” (ANP).

The main challenges to implementation were identified as the need for making pain management “*as important as safety*” (C), mandatory pain education focusing on “*models of care, the communication, speaking to the family, the explanation about things, the documentation*” (PN), “*more staff*” (PN), and “*more resources*” (C). Another suggestion was a shift in the culture, mindset, and working practices, all of which would help raise expectations about pain management:

“changing the culture that, if you work in a culture where nobody feels it is acceptable for a child to be, a young person to be in pain” (C).

## 4. Discussion

**Figure 1:**
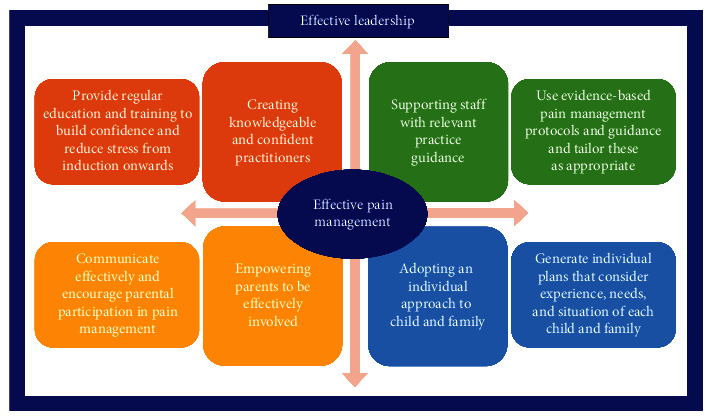
Framework for delivering effective pain management.

The intention of the study was to explore the relevance of five key elements and their potential for supporting effective management of children's pain and implementation of the framework into practice. Participants saw value in a framework for strategically supporting the necessary resources for pain management services and a structured means of informing and educating staff about pain. However, changes were suggested to make the framework more easily understood and to clarify what each element addresses (Figure 1). The interconnected elements focus on supporting staff, being confident, empowering parents, and adopting an individual approach; these are encompassed by a need for effective leadership and underpinned by education. This framework builds on the original interviews with 28 international pain practitioners, with the findings from 43 UK-based practitioners, and thus, it has a sound evidence-base. A framework such as this creates the potential for prompting conversations about the components underpinning effective children's pain management. The need for this is evident from the call from many participants who wanted the prioritisation of pain management mandated, as seen in other studies [18].

Although the various elements of the new framework are not in themselves truly innovative, what is innovative is the synergistic energy created by the amalgamation of the elements as a whole, encompassing practices that individually contribute to improve an area of challenge in the delivery of effective management of children's pain. Each individual element interacts with the other elements and combined are greater than the sum of their parts.

### 4.1. Creating Knowledgeable and Confident Practitioners, Supporting Staff with Relevant Guidance

Participants talked of the importance of knowledgeable and confident practitioners and felt that this could be achieved through ongoing and high-quality education. Some participants called for additional in-service education on children's pain, as seen in the findings from another research study [18]. This knowledge could be delivered at different levels depending on the experience, expertise, and requirements of the practitioners. There is little question that improved education can potentially improve pain practice. However, without a framework in which to structure this education, practitioners and educators could be missing out on opportunities to get the most out of limited education time. This became a focus of our thinking in terms of how different levels of education could be structured. The cognitive (knowledge-based) domain within Bloom's taxonomy of learning [19], updated by Forehand [20] consists of six levels. The first three (knowledge, understand, and apply) are relevant to all practitioners managing children's pain, albeit that the depth of knowledge will vary between practitioners from different disciplinary backgrounds, level of experience, and work setting (e.g., surgical ward or paediatric pain team). The second three (analyse, evaluate, and create) occur with practitioners operating at a higher level.

A recent study from Ghana suggests the need for pain education as a strategy to improve ineffective pain management practices [21]. However, there is a continuing lack of pain content in nursing curricula, particularly for undergraduate students [22–24]. Comprehensive pain assessment and management are essential to reduce the prevalence and burden of pain, and new strategies are required to support these changes.

There is also evidence that most healthcare professionals, including nurses, continue to exhibit inadequate knowledge and inappropriate attitudes toward the management of children's pain [25, 26]. Sufficient knowledge and positive attitudes of nurses and other healthcare professionals are thus required to improve the assessment and management of children's pain. A new framework, such as this paper presents, has the potential to meet this need.

Integration of Bloom's taxonomy [19] with the new framework offers a way forward for the promotion of effective pain management: in our study, education was seen as a fundamental way of sharing knowledge to support colleagues to deliver effective and knowledgeable pain management. This required both knowledgeable practitioners, educators, and specialists to deliver the education sessions but also sustained leadership and support from senior management to ensure that this education was accessed and shared widely so that the workforce had a necessary knowledge to deliver effective pain management. Knowledge alone is not enough, and there is a need to understand pain management and to apply it experientially over time, in order to grow in confidence in delivering effective pain management.

Building on these first three stages of Bloom's taxonomy [19], analyse, evaluate, and create reflect the capacity of a practitioner for continuous practice development/improvement building from the ongoing day-to-day application of existing pain management practices. Recognising areas of practice that need to be improved is evident within the analyse stage. An analytical practitioner is able to explain and integrate the principles of pain management for an individual child and confidently inform and involve parents. A practitioner capable of considering the effectiveness of pain management strategies across a ward would be demonstrating that they can evaluate practice and would be critically considering why a pain management strategy was not working. Create is evident when new pain management practices are being developed or when a practitioner is able to appropriately adapt a pathway or protocol to meet a child's specific (often complex) needs.

Ideally, educational input would support practitioners to extend their knowledge, understanding, and practice in line with clinical expertise and then develop their capacity to analyse, evaluate, and create.

Participants also talked of pathways and guidelines as being key to effective pain management. They saw the framework as having a part to play in providing guidance. However, they were realistic that guidance, protocols, and pathways relied on people accessing and implementing them. There is now a wealth of well researched and clear pain management guidance [5, 6], pain pathways [27], care bundles [28], and pain assessment tools that support effective pain management that this framework could link to. However, it is clear from another research that implementation is by no means simple, and uptake of guidance is often patchy. Leadership is noted as an essential component in effectively translating guidance into practice [29].

### 4.2. Adopting an Individual Approach to the Child and Family and Empowering Parents to be Effectively Involved

The participants were clear that children deserved and needed individualised approaches to their pain management in order to generate optimum care. The child's and parents' individual context should be considered to ensure that an individualised plan is developed to meet that particular child's needs at that time. Tailored or individualised pain management for children needs to encompass tailored analgesic medication [30] and nonpharmacological approaches that manage anxiety and pain [31, 32].

Although some participants were cautious about raising parents' expectations, most talked of this as a key element to effective pain management. The skill lies in how practitioners engage and communicate with parents and the information they share with them. Preparation and information giving were identified as key components. As with other studies [33], we identified the need for better communication and information to enable parents to effectively participate in managing their child's postoperative pain. Where parents (and children) are given information on pain management, studies have shown that this results in satisfaction with care [9]. However, a lack of communication, information provision, and poor negotiation of roles can result in missed opportunities for nurses to work in partnership with parents [11]. Sometimes, information provision can be inconsistent, resulting in parents having sufficient information about some aspects (e.g., the surgical procedure) but not others (e.g., pain medication) [31]. Clearly empowerment can only really be achieved with consistent and high-quality information that supports parents' expectations of good care and gives them the confidence to complain about poor pain management [10, 34]. Paradoxically, research has found that strategies to improve pain management and encouraging greater engagement from parents can result in more complaints from parents [14]; this may result from parents being more informed, confident, and having higher expectations of what can be achieved. Empowering parents makes considerable sense, as they are their child's most consistent caregivers, and therefore an obvious source of personalized knowledge to assist in identifying and implementing strategies to optimize better pain care [35]. The potential for alerting parents to ask about their child's pain via posters as part of a visible campaign within the hospital is one way that information could reach parents.

## 5. Conclusion

The framework offers practitioners an overarching approach that can be used as a catalyst to address a range of resistant challenges identified in this study, by promoting efficient use of resources and targeting pain management education.

The framework has the potential to facilitate the development of skilled confident pain practitioners who empower parents to engage in their child's pain care. A follow-on study is planned to ascertain the views of parents in relation to their engagement in the management of their child's pain.

As a part of the dissemination work associated with the study, the team has created infographic posters about the framework, an animation [https://youtu.be/9r3XMKcFERk] that provides an overview of the key aspects of the framework. A free online course is being developed, which will integrate the cognitive domain of Bloom's taxonomy to facilitate learning.

## Data Availability

The data used to support the findings of this study have not been made available because the data are anonymised interview transcripts and will be made available from the corresponding author upon request.

## Supplementary Materials

Supplementary Materials Concise description: format of the focus group interview including questions.
