# Supplementary material for: Developing a Framework to Support the Delivery of Effective Pain Management for Children: An Exploratory Qualitative Study
Source: Pain Res Manag. 2020 Oct 28;2020:5476425. doi: 10.1155/2020/5476425 (PMC7641711; doi:10.1155/2020/5476425)
Supplement: Supplementary Materials — Concise description: format of the focus group interview including questions. [file 5476425.f1.docx]

*
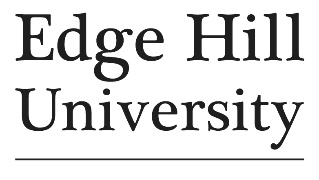

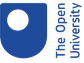
*
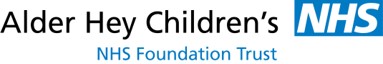


**Focus Group Interview Schedule**

Hello,

Thank you for agreeing to take part in this Focus Group interview. Our aim today is to gain your views on the pain model that has been developed to try to improve the management of children’s pain and check what you consider the implications are of implementing the model in practice.

Do you have any questions at this point?

(Any questions posed are answered)

If you are satisfied that you understand what the study is about and are still willing to participate I would like to ask you to sign a consent form.

(Interviewer explains consent form, answers any questions and gains written consent and permission to record the interview)

Thank you for agreeing to take part, if at any point you change your mind during the interview you are free to withdraw.

Before we begin I would like to clarify the need for confidentiality. It is important that we are all able to contribute so it is best to not speak over people and wait your turn, as we want to hear what everyone has to say. You may find in our discussion that you give examples from practice, if so please remember not to mention patients’ names or other colleagues’ names.

I will be your moderator today asking the questions and keeping the conversation on track and my colleague will be the facilitator, who is supporting me.

We will ask you questions about your views of implementing the pain model and the point of the focus group is to be able to gain everyone’s perspective and at the same time discuss what you hear during the conversation.

Explanation of the pain model using the following text:

Joan Simons (the Principal Investigator of this study) interviewed practitioners in different countries about what they found to be the key components of effective management of children’s pain. The findings showed that a sequence of interrelated components appeared to contribute and enhance the effectiveness of the management of children’s pain (see Figure 1).

**Figure 1: Components that support management of children’s pain (Simons, 2015)**

This ‘model’ has not been tested in any way. The purpose of this study is to explore with nurses and other professionals how practical such an approach is in practice.

Broad topics to be covered in the focus group are:

1. **How could the following aspects of the model be implemented in your area of practice:**
2. Distributed pain management leadership with vision
3. Effective pain management with less stress
4. (Pain management) Delivered with confidence
5. Individual approach to child and parent
6. Raising parents’ expectations of effective pain management

**2. In your opinion how realistic is the model as a whole?**

**3. What would need to change in your place of work to facilitate the implementation of the model?**

**4. Do you have anything else to add?**

(These questions are guiding ones and will not necessarily be asked in order as the aim is to facilitate a  reasonable conversation so there is a need to be responsive to the discussion whilst also guiding the conversation)

**At the end of the interview:**

Thank you for your time today and for contributing to this study. We very much appreciate your involvement.

Do you have any questions for me or any concerns about the interview?

(If not, the interview ends. If questions or concerns are raised the interviewer will answer them / address them at this point.)
